# CytroCell Micronized Cellulose Enhances the Structural and Thermal Properties of IntegroPectin Cross-Linked Films

**DOI:** 10.1021/acsabm.2c00658

**Published:** 2022-10-07

**Authors:** Antonino Scurria, Mario Pagliaro, Giuseppe Pantaleo, Francesco Meneguzzo, Francesco M. Giordano, Rosaria Ciriminna

**Affiliations:** † Istituto per lo Studio dei Materiali Nanostrutturati, CNR, via U. La Malfa 153, 90146 Palermo, Italy; ‡ Dipartimento DICEAM, Università degli Studi “Mediterranea” di Reggio Calabria, Via Graziella, Loc. Feo di Vito, 89122 Reggio Calabria, Italy; § Istituto per la Bioeconomia, CNR, via Madonna del Piano 10, 50019 Sesto Fiorentino, Florence, Italy

**Keywords:** film, CytroCell, cellulose, IntegroPectin, biocompatible, pectin

## Abstract

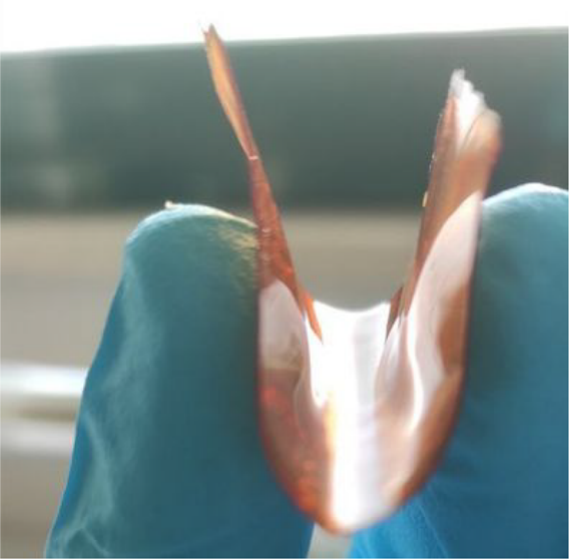

Added to grapefruit IntegroPectin in solution, the micronized cellulose CytroCell, coproduct of the IntegroPectin extraction via hydrodynamic cavitation, enhances the structural and thermal properties of the resulting cross-linked composite films. The films become strong but remain highly flexible as no transition glass temperature is observed, whereas the thermal properties are substantially improved. No organic solvent, acid, or base is used from the extraction of the pectin and cellulose biopolymers through filming their nanocomposites, thereby establishing a completely green route to a class of bio-based 2D films (and 3D scaffolds) with numerous potential applications in regenerative medicine, in tissue engineering, and in the treatment of infections.

## 1 Introduction

Besides being the most valued food hydrocolloid,^1^ pectin is a uniquely versatile polysaccharide whose remarkable chemical and biological properties originate numerous advanced applications, well beyond its use as gelling agent and thickener in the food and beverage industries.^2^

Dissolved in water pectin easily forms films by simple solution casting followed by water evaporation. These biocompatible and biodegradable films have recently been investigated for multiple aims, from drug release^3^ through food packaging.^4^

Remarkably, chemists started to investigate the use of pure and cross-linked (with Ca^2+^ ions) pectin films using glycerol as plasticizer in 1949, when USA-based chemists reported that transmission rates for the permeation of water vapor through calcium pectinate films 40 μm thick are 1400–4300 g m^–1^ day^–1^, similar to cellophane.^5^

Nearly one century later, quantum chemistry calculations suggest that H_2_O molecules strongly adsorb on the pectin film thanks to formation of hydrogen bonding between the −OH group of galacturonic acid (GA) moieties and GA and H_2_O, and eventually slowly diffuse through the film to the other side.^6^ Oxygen molecules, in their turn, can only diffuse via Knudsen diffusion through micropores or microdefects on the film surface.

In 1949 the era of synthetic plastics was opening. The interest for bio-based polymers vanished for several decades. The results achieved with pectin-based films clearly investigated as “edible” alternatives to cellophane for food packaging,^5^ remained virtually ignored for nearly 50 years.

With the advent of the 1990s, however, this pioneering work was rediscovered. Driven by the environmental (and health) problems posed by non-biodegradable plastics, the interest for bio-derived polymers rose again. In a few years, chemists quickly discovered that the mechanical, thermal, and barrier properties of pectin-based films can be largely improved by adding (“blending”) other polymers. Examples include starch,^7^ ethylcellulose,^8^ poly(vinyl alcohol),^9^ hydroxypropyl methylcellulose,^10^ and gelatin.^11^

Research further intensified with the emergence of the bioeconomy, since pectin is commercially produced from two agriculture byproducts, waste citrus peel and apple pomace.^12^

In 2017, Iran-based researchers first reported the remarkable reinforcing effect of cellulose nanocrystals (CNCs) on the mechanical, thermal, and barrier properties of pectin-based films prepared using solution casting evaporation method.^13^ It is enough to add a 5% CNC concentration to increase the film tensile strength up to 84%, and decrease water vapor permeability by 40%.

In general, cellulose fibers reduce the permeability to water of bioplastics.^14^ The lower the cellulose fiber size the better, because by reducing fiber size, a high surface to volume ratio of the filler in the matrix is achieved. Similarly, nanocellulose added to biopolymers greatly enhanced their tensile strength due to formation of a stiff continuous network of hydrogen-bonded cellulose nanofibers,^15^ and to strong interaction between pectin carboxyl and hydroxyl groups and cellulose hydroxyls through interfacial hydrogen bonds and ionic interactions.^16,17^

We now report the first outcomes of structural and thermal investigation of new cross-linked citrus “IntegroPectin” pectic films functionalized with the “CytroCell” micronized cellulose. Both biomaterials were obtained in one pot processing via hydrodynamic cavitation (HC) industrial biowaste of grapefruit in water only on semi-industrial scale (30 kg of biowaste in 120 L of water). IntegroPectin^18^ is isolated via mild (freeze) drying of the aqueous extract, whereas the CytroCell^19,20^ insoluble cellulosic fraction is simply recovered by filtration followed by mild drying.^20^

## 2 Experimental Section

All films (PCF-1, PCF-2, and PCF-3) were prepared by starting from a solution of grapefruit IntegroPectin in ultrapure water obtained from a Thermo Scientific Barnstead Smart2Pure water purification system (Thermo Fisher Scientific, Waltham, MA, USA). Both grapefruit IntegroPectin and grapefruit CytroCell were obtained in one pot via HC-based extraction using a procedure described in detail elsewhere.^21^

**Scheme 1:**
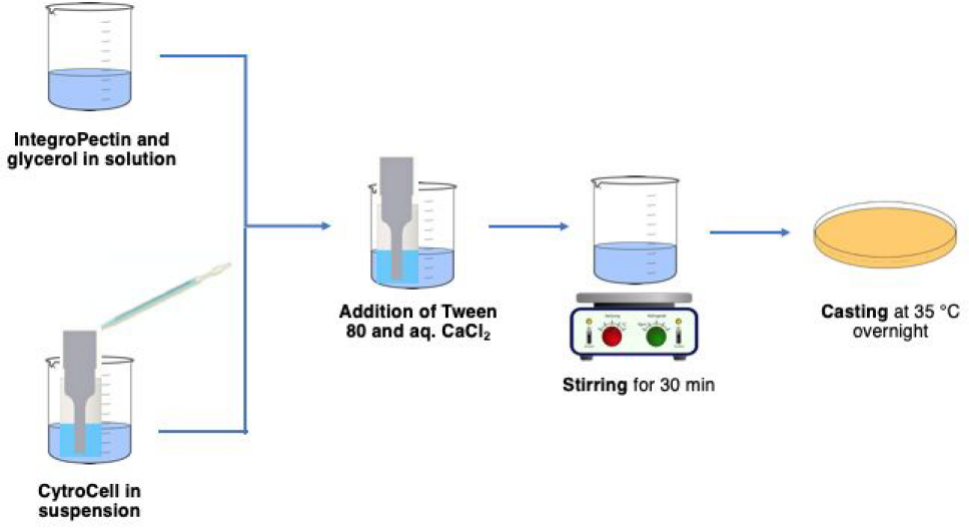
Preparation of Grapefruit IntegroPectin Cross-Linked Pectin Films Functionalized with Grapefruit CytroCell Micronized Cellulose

Scheme 1 outlines the simple film preparation process.

Table 1 summarizes the amounts of biomaterials and glycerol plasticizer used to prepare the films cross-linked with aqueous CaCl_2_.

**Table 1:** Components Used to Prepare the CytroCell–IntegroPectin Films Cross-Linked with Aqueous CaCl_2_

cross-linked film	IntegroPectin (g)	CytroCell (g)	glycerol (g)
PCF-1	1.5	0.1	0.3
PCF-2	1.5	0.1	0.2
PCF-3	1.5	0.2	0.3

In detail, the PCF-1 film was prepared adding a 100 mL round balloon with 30 mL of water, 1.5 g of IntegroPectin, and 300 mg of glycerol (≥99.5%, Sigma-Aldrich Italia, Milan, Italy). The mixture was left under stirring at room temperature for 20 min at 500 rpm. A 60 mL round-bottom vial was added with 30 mL of water and 100 mg of grapefruit CytroCell. The sample was sonicated for 5 min with a Sonopuls HD 4100 homogenizer (Bandelin Electronic, Berlin, Germany) operated at 20% amplitude. The latter solution was thus added with the CytroCell suspension dropwise under moderate magnetic stirring. The resulting mixture underwent sonication for 5 min under the same conditions mentioned above, after which the homogenized mixture was added with 100 mg of polyethylene glycol sorbitan monooleate (Tween 80, Sigma, Merck KGaA, Darmstadt, Germany) under magnetic stirring at 600 rpm. The mixture was left under stirring for 10 min, after which it was added with 5 mL of 2% (w/v) CaCl_2_ (anhydrous, Carlo Erba Reagenti, Rodano, Italy) aqueous solution. After 30 min, agitation was stopped and the mixture cast in two Petri dishes (8 cm diameter). The films were obtained by solvent casting, leaving the Petri dishes in a ventilated (10% ventilation) UF 30 oven (Memmert, Schwabach, Germany) overnight at 35 °C.

The PCF-2 film was prepared using a lower amount (200 mg) of glycerol, whereas PCF-3 was prepared by doubling to 200 mg the amount of CytroCell, retaining the original amount (300 mg) of glycerol.

Thermogravimetric analysis (TGA) was performed using a TGA/DSC1 STARe System (Mettler Toledo, USA). Measurements were carried out using ∼15 mg of ground samples in a 70 μL crucible at a heating rate of 10 °C/min from 30 to 1100 °C with a 50 mL/min nitrogen flow. One TGA per sample was acquired.

The samples were analyzed by a D5005 X-ray diffractometer (Bruker AXS, Karlsruhe, Germany) operating at 40 kV and 30 mA to obtain the diffraction profile at 0.60°/min acquisition rate over a 5.0°–35.0° 2θ range with increment 0.05°. The X-ray radiation was generated via a copper (Kα) anode and made monochromatic via the instrument’s secondary monochromator.

The FTIR spectra were recorded via the attenuated total reflection (ATR) sampling technique using the multi-reflection ZnSe ATR sampling module of the Alpha FTIR spectrometer (Bruker, Billerica, MA, USA).

## 3 Results and Discussion

**Figure 1:**
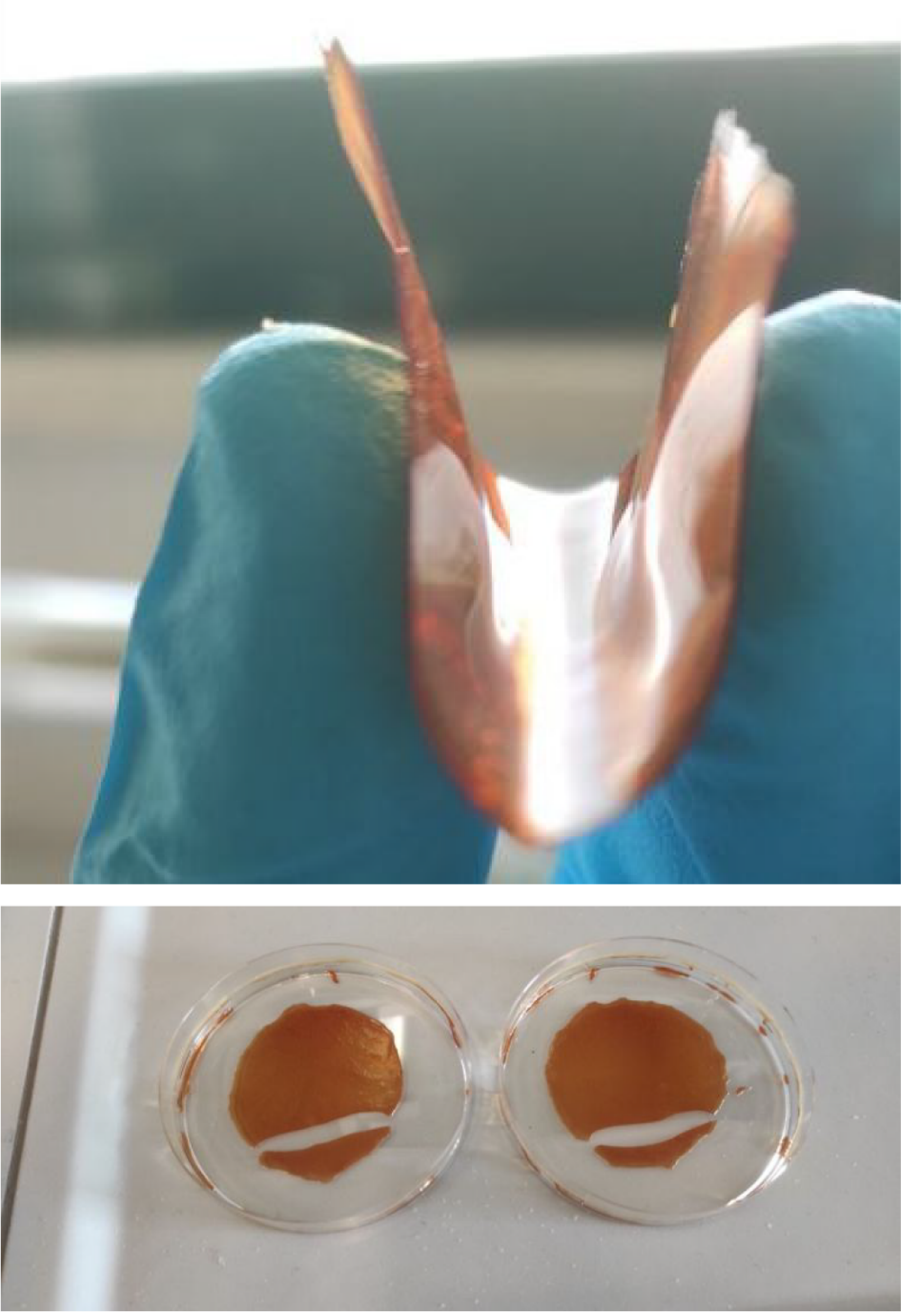
PCF-3 (top) and PCF-2 (bottom) films.

Figure 1 shows the PCF-3 and PCF-2 films. As shown in Figure 1, PCF-3 is at the same time mechanically strong and highly flexible, a particularly important structural feature in light of forthcoming biomedical applications of these films.

**Figure 2:**
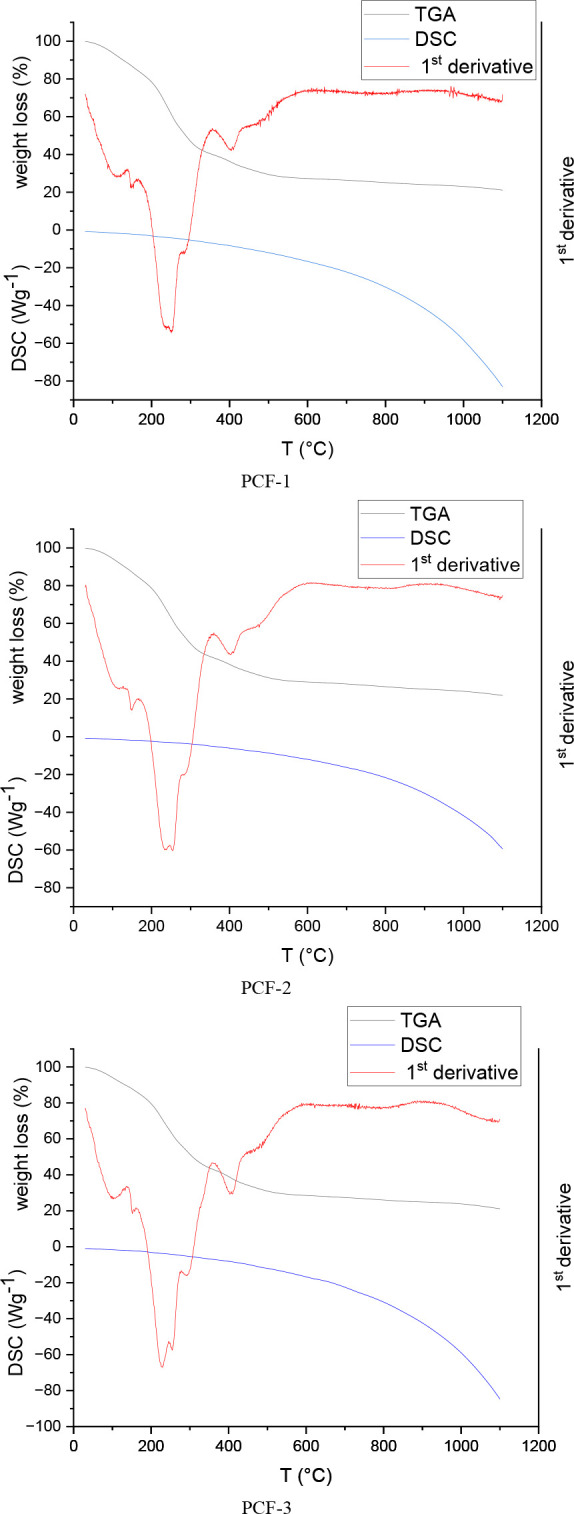
TGA, DTG, and DSC curves for the three cross-linked IntegroPectin/CytroCell films: PCF-1 (6.67 wt % CytroCell, 20 wt % glycerol); PCF-2 (6.67 wt % CytroCell, 13.3 wt % glycerol); and PCF-3 (13.3 wt % CytroCell, 20 wt % glycerol).

The TGA, DTG, and DSC curves are displayed in Figure 2 for all of the films.

Proportional to the rate of sample decomposition, the derivative thermogravimetry curve (DTG, the first derivative of the TGA curve with regard to time for which the weight loss is differentiated with respect to time and then the as-obtained values are plotted against the temperature) indicates that for the PCF-1 and PCF-2 films the pectin decomposition rate reaches a maximum at ∼240 °C and is thus independent of the amount of glycerol used as plasticizer. We remind that glycerol acts as a plasticizer by reducing the intra- and intermolecular forces among the polysaccharide chains, improving the flexibility and water absorption capacity of pectin-based films.^21^

Due to pectin decomposition, this weight loss originates from cracking of the saccharide rings in pectin.^22^ On the other hand, for the PCF-3 film produced using twice the amount of CytroCell, the pectin decomposition rate reaches a maximum at 220 °C, with a faster and more significant weight loss at 100 °C due to the higher amount of adsorbed H_2_O molecules. In addition, the two peaks at 280 and 400 °C are significantly more pronounced.

Showing first evidence of the significant physical protective action of the IntegroPectin pectin chains stabilizing the incorporated cellulose fibrils, the peak at 280 °C, corresponding to cellulose dehydration to form dehydrocellulose, is observed at significantly higher temperature than that (250 °C) of CNC.^23^

A similar remarkable shift was observed for the second main thermal degradation pathway of cellulose, namely, depolymerization, which in the case of grapefruit CytroCell alone was at 337 °C.^19^

In the case of grapefruit IntegroPectin/CytroCell films, cellulose depolymerization takes place at ∼400–405 °C. We ascribe the ability of the IntegroPectin polymeric chains to protect and stabilize the incorporated cellulose fibrils to the strong hydrogen bonds forming between the free carboxylic acid groups of low esterified grapefruit IntegroPectin (degree of esterification of 14%)^24^ and the grapefruit CytroCell cellulose fibrils consisting of highly ramified microfibrils with diameter ranging from 500 to 1000 nm.^19^

The DSC thermograms of all films show a broad endothermic curve within the whole temperature range studied, suggesting the possible non-existence of a glass transition temperature (to verify this hypothesis, it would be necessary to carry out the DSC measurements in equipment separate from the TGA and with a temperature range up to the beginning of the material’s degradation) and retention of the thermoplastic behavior observed at room temperature (Figure 1, top) for the cross-linked grapefruit IntegroPectin/CytroCell films even at a CytroCell load of 13.3 wt %.

**Figure 3:**
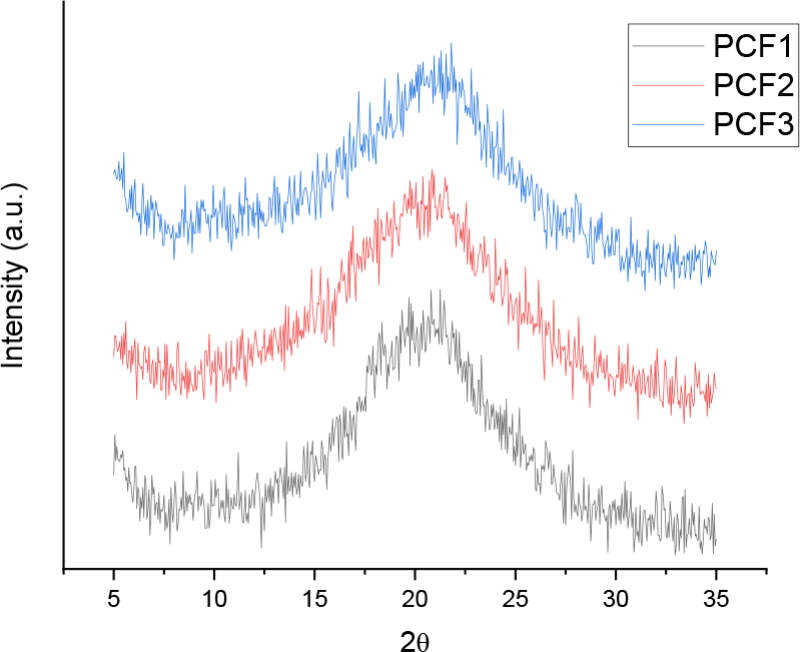
XRD patterns for the three cross-linked grapefruit IntegroPectin/CytroCell films: PCF-1 (6.67 wt % CytroCell, 20 wt % glycerol); PCF-2 (6.67 wt % CytroCell, 13.3 wt % glycerol); and PCF-3 (13.3 wt % CytroCell, 20 wt % glycerol).

The XRD patterns of the three cross-linked grapefruit IntegroPectin/CytroCell composite films in Figure 3 are nearly identical. The broad halo peak at 2θ = ∼21° observed for all of the films indicates the semicrystalline arrangement of the pectin chains partly ordered in a helical structure,^25^ with Ca^2+^ ions creating a water-insoluble cross-linked gel structure containing a 3D network of Ca^2+^ ion-bridged dimers to induce so-called “egg-box” junction zones.^26^

Reducing the amount of glycerol (going from PCF-1 to PCF-2) or doubling the amount of micronized CytroCell cellulose (going from PCF-1 to PCF-3) did not affect the diffraction peak maximum, thereby confirming the existence of an optimal glycerol concentration of the plasticizer beyond which further addition does not affect the polysaccharide molecular arrangement.^27^

The fact that increasing the amount of CytroCell does not alter the XRD profile shows once again how the poorly crystalline grapefruit cellulose microfibrils (grapefruit CytroCell has a crystallinity index of 0.36 only)^20^ smoothly interpenetrate the mostly amorphous IntegroPectin chains.

**Figure 4:**
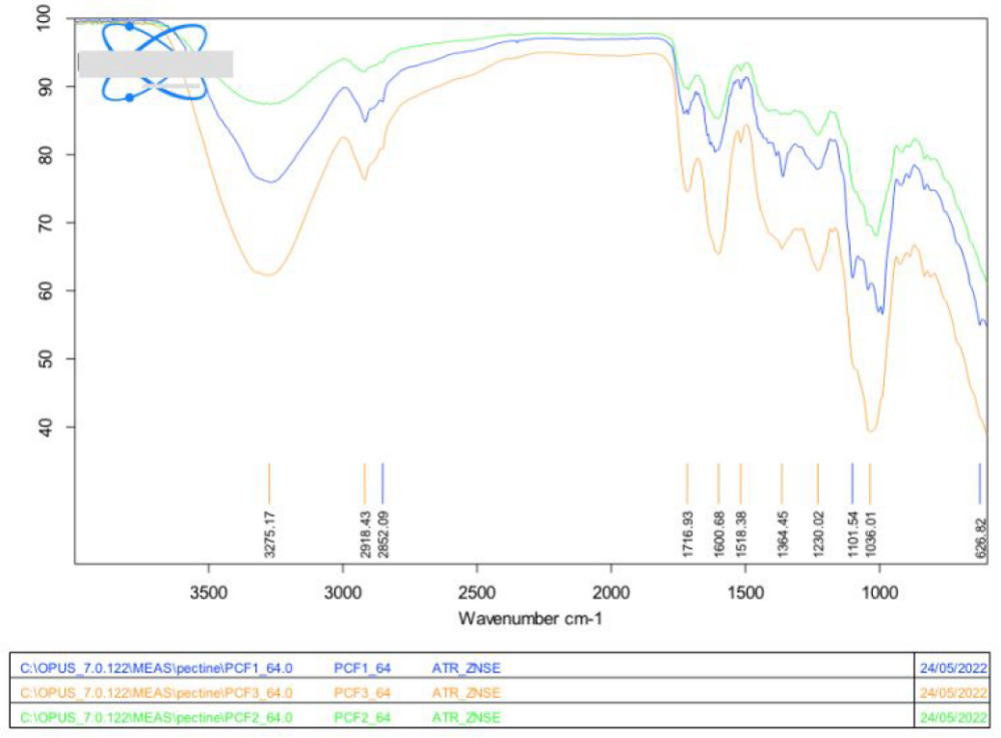
XRD patterns for the three cross-linked grapefruit IntegroPectin/CytroCell films: PCF-1 (6.67 wt % CytroCell, 20 wt % glycerol); PCF-2 (6.67 wt % CytroCell, 13.3 wt % glycerol); and PCF-3 (13.3 wt % CytroCell, 20 wt % glycerol).

Figure 4 shows that all of the typical FTIR absorption bands of pectin and cellulose are observed for each film, with the broad signal of the O–H stretching mode at 3275 cm^–1^ being a broad signal significantly more pronounced in the PCF-3 film, in agreement with the highest amount of hydrophilic cellulose in the composite.

In agreement with the FTIR spectrum of grapefruit IntegroPectin,^24^ also the composite grapefruit IntegroPectin/CytroCell films showed one large ν_s_(C=O) stretching mode signal centered at ∼1716 cm^–1^ of carboxylate groups and another at ∼1600 cm^–1^ due to ν_as_(COO^–^).^28^

**Figure 5:**
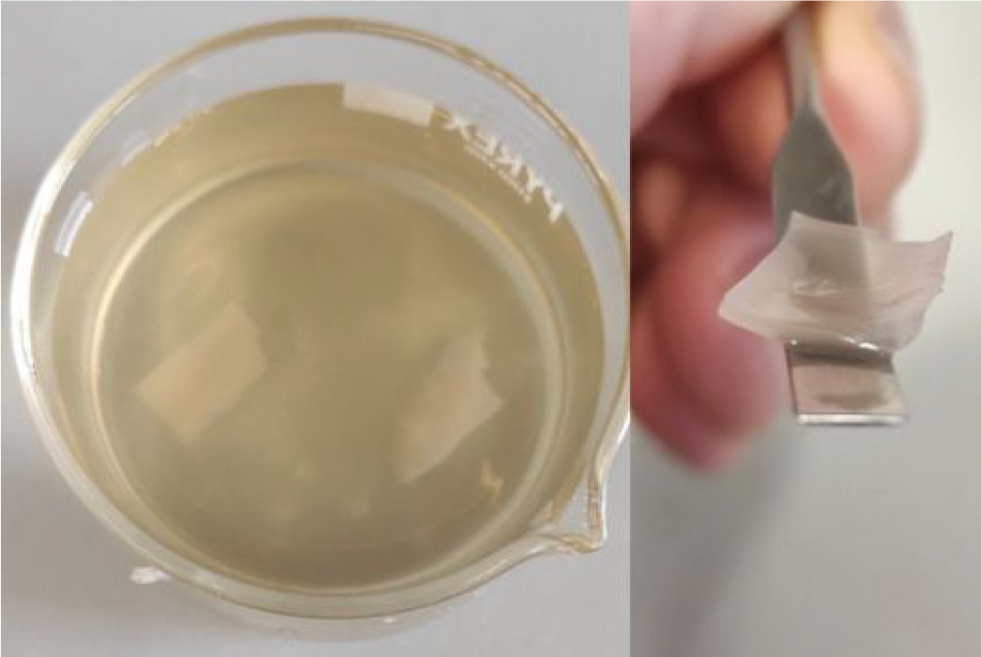
PCF-1 film immersed in water (left) and retrieved (right) after 10 days of immersion in water.

Added to water, the films do not dissolve and only moderately swell after several days of immersion in water (Figure 5), eventually leaching in solution most water-soluble adsorbed molecules.

The high stability in water also stems from the fact that cross-linked pectin films from pectins of low DE such as grapefruit IntegroPectin are more stable due the a greater Ca^2+^ ion content and thus more cross-links, yielding a more stable structure.^29^

The sustained release of bioactive molecules from similar (but cellulose-free) IntegroPectin cross-linked films has been recently quantified for several citrus biophenols and partly found responsible for the powerful antimicrobial activity of said films.^30^

## 4 Conclusions

In conclusion, we have discovered that (i) IntegroPectin/CytroCell films can be readily produced using a completely green biomaterial production route using only a bio-based plasticizer (glycerol), a bio-based surfactant (polysorbate 80), water, and CaCl_2_; and that (ii) the addition of micronized cellulose CytroCell to cross-linked grapefruit IntegroPectin films largely enhances the structural and thermal properties of the IntegroPectin cross-linked films.

No organic solvent, acid or base is used from the extraction of the new pectin and cellulose biopolymers from citrus juice biowaste, through filming their nanocomposites, thereby establishing a completely green route to a new class of biocompatible and highly bioactive 2D films (and 3D scaffolds) with numerous potential applications. First demonstrated with grapefruit, these results are general and can be extended to all other citrus juice biowastes, including those of orange and lemon.

Use of these nanocomposite films is anticipated in regenerative biomedicine and in the treatment of infections. Similar films obtained without cellulose, indeed, were recently shown to be powerful antimicrobials, capable of killing harmful clinical pathogens of *Klebsiella pneumoniae*.^30^ Pectin, furthermore, is used by pharmaceutical and biomedical companies to produce wound dressings and ostomy care products.^2^

Currently, pectin-based 2D films and 3D structures are widely investigated in tissue engineering for the fabrication of functional scaffolds via bioprinting.^31^ The main limitations to widespread utilization of pectin as bio-ink to produce bio-based films and scaffolds are the poor barrier and poor mechanical properties of pectin-based coatings.

Nanocellulose, chiefly in the form of cellulose nanocrystals, has been successfully used to develop mechanically strong and water resistant pectin-based composite films and composites,^13^ suitable for the controlled release of important drugs such as hydroxychloroquine,^32^ or even as biodegradable and biocompatible light lenses due to excellent optical properties.^33^

Compared to expensive CNC,^34^ however, CytroCell micronized cellulose is obtained in one pot, at negligible cost, directly along with the same highly bioactive pectin (IntegroPectin)^18^ used to produce the composite films.

## Notes

All experimental data are available by contacting the Corresponding Author.
